# The arrow and the target—description of the “Robin Hood” technique for fixation of tibial spine avulsion fractures: a technical note

**DOI:** 10.1007/s00590-026-04915-7

**Published:** 2026-08-08

**Authors:** Karl-Heinz Frosch, Robinson Esteves Pires, Harm Hoekstra, Gustavo Waldolato Silva, Rafael Chagas Silva, Danilo Taype Zamboni, Vincenzo Giordano

**Affiliations:** 1 Department of Trauma and Orthopaedic Surgery, University Medical Center Hamburg-Eppendorf, Hamburg, Germany; 2 Department of Trauma Surgery, Orthopaedics and Sports Traumatology, BG Klinikum Hamburg, Hamburg, Germany; 3 Department of the Locomotor System, Federal University of Minas Gerais, Belo Horizonte, Brazil; 4 Orthopaedic Trauma Unit, Felicio Rocho Hospital, Belo Horizonte, Brazil; 5 Department of Trauma Surgery, University Hospitals Leuven, Leuven, Belgium; 6 Department of Development and Regeneration, KU Leuven, Leuven, Belgium; 7 Orthopaedic Trauma Unit, Felicio Rocho Hospital, Belo Horizonte, Brazil; 8 Orthopaedic Trauma Unit, Hospital Italiano de Buenos Aires, Buenos Aires, Argentina; 9 Miguel Couto Hospital, Rio de Janeiro, Brazil

**Keywords:** Knee fractures, Tibial plateau fractures, Intercondylar eminence fractures, Tibial spine fractures

## Abstract

Avulsion fractures of the tibial spine, also known as the tibial eminence, are predominantly described in the pediatric population and uncommon in adults. In the presence of a tibial spine fracture associated with a fracture of the proximal meta-epiphyseal tibia, management of the spine fragment remains controversial. The objective of this technical note is to describe the Frosch technique termed the “Robin Hood technique”, which consists of fixation of the tibial spine fracture with screws inserted in a retrograde fashion in cases of combined fractures of the tibial spine (due to avulsion of the anterior cruciate ligament) and fractures of the proximal meta-epiphyseal area of the tibia.

## Introduction

Tibial spine fractures correspond to avulsion of the tibial insertion of the anterior cruciate ligament (ACL) and occur more frequently in children and adolescents due to the biomechanical characteristics of the immature skeleton [1, 2]. In adult patients, they are less frequent and, when present, usually associated with higher-energy mechanisms and combined intra-articular injuries [3]. 

The Meyers and McKeever modified by Zaricznyj classification remains the reference for understanding these injuries according to the degree of fragment displacement and the presence of comminution [1]. In general, non-displaced tibial spine fractures (type I of the Meyers and McKeever classification modified by Zaricznyj are treated with knee in extension or slight flexion (20°–30°) immobilization for a period of 6 to 12 weeks, whereas displaced fractures tend to receive surgical treatment aimed at restoring the functional length of the ACL and the articular stability of the knee [4]. 

In adult tibial plateau and proximal meta-epiphyseal tibial fractures associated with tibial spine injuries, the role of tibial spine fixation remains less defined, particularly regarding indications and optimal surgical technique [2, 3]. Flexion-valgus trauma is strongly associated with ACL injury and lateral meniscal tears due to posterolateral plateau depression and subluxation. Wang et al. [5] reported 83% ACL injuries in posterolateral tibial plateau fractures, including 38% avulsion fractures and 25% complete in-substance tears. In contrast, flexion-varus tibial plateau fractures, ACL injuries occur in up to 91% of cases, most commonly as avulsion fractures (85%), due to rotational and varus forces [6]. 

Bony avulsion injuries ideally should be fixed at the time of fracture fixation. Several surgical techniques for tibial spine avulsion fractures have been described, including interfragmentary compression or osteosynthesis with antegrade screws (cannulated or solid), repair with wires and transosseously sutures or anchors, performed through traditional open, minimally invasive, or arthroscopic approaches [4, 7]. Arthroscopic techniques using suture augmentation and adjustable tension devices have also been described in recent technical notes [7, 8]. 

This technical note aims to expand the surgical decision-making planning by introducing a simple and useful fixation technique for tibial spine fractures associated with standard reconstruction of proximal tibial meta-epiphyseal and tibial plateau fractures.

## Methods

The study was conducted in accordance with the Declaration of Helsinki and was approved by the institutional Research Ethics Committee.

### Indication

The Robin Hood retrograde fixation technique for tibial spine avulsion fractures is indicated in the following situations:

- Skeletally mature patients presenting anterior tibial spine fractures associated with proximal meta-epiphyseal fractures of the tibia.

## Contraindications

- Extensive comminution may preclude the use of this technique and should be considered as a contraindication.
- Fracture fragments that cannot be anatomically reduced using the described technique are not suitable for fixation with this method. Especially if soft tissue interference prohibits proper reduction. In selected cases, reduction may be achieved through percutaneous maneuvers or limited open approaches using auxiliary instruments, such as an ACL guide, arthroscopy probe, or delicate forceps, under arthroscopic or fluoroscopic guidance.

## Technique description

### Preoperative planning

Radiographs in orthogonal planes and computed tomography (CT) are mandatory components of the diagnostic work-up. CT is essential for preoperative planning, allowing assessment of fragment size, displacement, morphology, and degree of comminution, which are critical factors in determining the suitability of screw fixation.

MRI is not routinely obtained in all patients with tibial spine fractures at our institution. Instead, its use is selective and guided by the injury mechanism and the suspicion of associated soft-tissue injuries that may influence treatment planning.

Specifically, we request MRI in cases involving a hyperextension mechanism, when there is concern for meniscal root injury or meniscal entrapment/interposition within the fracture site. MRI is also obtained in fracture-dislocation injuries, in which the likelihood of associated ligamentous injuries is substantially higher, including injuries to the medial or lateral collateral ligament complexes and the posterolateral corner [8, 9]. 

### Patient positioning

Patient positioning is determined by the approach selection to the tibial plateau fracture, with the prone position utilized for posterior approaches (tibial spine fixation from posterior to anterior) and the supine position for anterior approaches (tibial spine fixation from anterior to posterior).

The C-arm fluoroscope should be positioned contralateral to the surgeon.

### Surgical technique

The use of a pneumatic tourniquet is individualized according to surgeon preference and surgical planning.

Initially, fixation of the proximal tibial metaphyseal fracture according to the injury pattern is recommended in order to avoid potential conflict among implant trajectories.

Under fluoroscopy, it must be confirmed that the tibial spine fragment is reduced and that there is no intra-articular blockage. Attention should be paid to possible interposition of the intermeniscal ligament within the fracture site, as this ligament crosses between the medial and lateral menisci immediately anterior to the intercondylar eminence and may act as a mechanical obstacle to fragment reduction [4]. This situation is more frequent when there is an associated lateral meniscus injury [9, 10]. 

In cases of anterior displacement of the tibial spine fragment, reduction can be performed through a posterior arthrotomy in the prone position (posteromedial approach) or through an anteromedial arthrotomy in the supine position. Anatomical reduction is achieved using a small raspatory or a Kelly clamp prior to definitive fixation. An arthroscopic hook or drill guide may be used in selected cases when additional control of the fragment is required. The principles of reduction and the instruments used are the same regardless of the direction of tibial spine fragment displacement. However, the surgical window selected for introducing the reduction instruments and, when necessary, patient positioning may be modified to optimize visualization and facilitate anatomic reduction. Because fracture morphology and displacement patterns vary among patients, the reduction strategy should be individualized based on the specific fracture characteristics and intraoperative findings. If the described technique is used with the patient in the prone position using a posteromedial approach, the aiming device (drill guide) is introduced into the joint through the posterior interval between the ACL and PCL. The tibial spine fragment can be reduced using the hook of the drill guide. The aiming device then allows fixation of the fragment with K-wires inserted through the drill guide sleeve. These K-wires subsequently serve as guidewires for retrograde insertion of cannulated screws.

A guide wire (in the case of cannulated screws), drill, or Kirschner wires are introduced into the proximal tibial metaphyseal region using a retrograde trajectory, from either anterior to posterior or posterior to anterior (depending on the patient positioning), with an anterior entry point 3–7 cm from the articular surface. Subsequently, fixation of an ACL avulsion fracture can be performed using either a posterior approach, applied for the treatment of a flexion-type tibial plateau fracture.

In our preferred technique, screw fixation is performed through the posterior cortex. In selected cases requiring buttress plate fixation, screws may be inserted through the plate when the implant trajectory is compatible with tibial spine fixation.

In our practice, implant selection is primarily based on fragment size. For avulsed fragments measuring > 15 mm, we prefer fixation with two screws, most commonly 2.7-mm solid cortical screws, as they provide stable fixation while minimizing the risk of fragment comminution. For fragments measuring < 15 mm, we generally prefer fixation with a single 3.5-mm screw, provided that stable fixation can be achieved without compromising the integrity of the fragment. Fragment morphology and bone quality should always be considered, and fracture fragments that cannot be securely fixed with screws should be treated with an alternative fixation method, such as suture fixation.

To avoid intra-articular screw penetration, preoperative CT is used to assess fragment morphology and plan the screw trajectory. During fixation, guidewire and screw positioning are sequentially verified under fluoroscopy, ensuring that the trajectory remains within the tibial spine fragment and below the subchondral bone. Standard anteroposterior and lateral fluoroscopic views are used, with additional oblique views when necessary to confirm the trajectory and exclude intra-articular penetration. Final screw length is selected only after confirmation of satisfactory positioning on multiple fluoroscopic views.

**Fig. 1:**
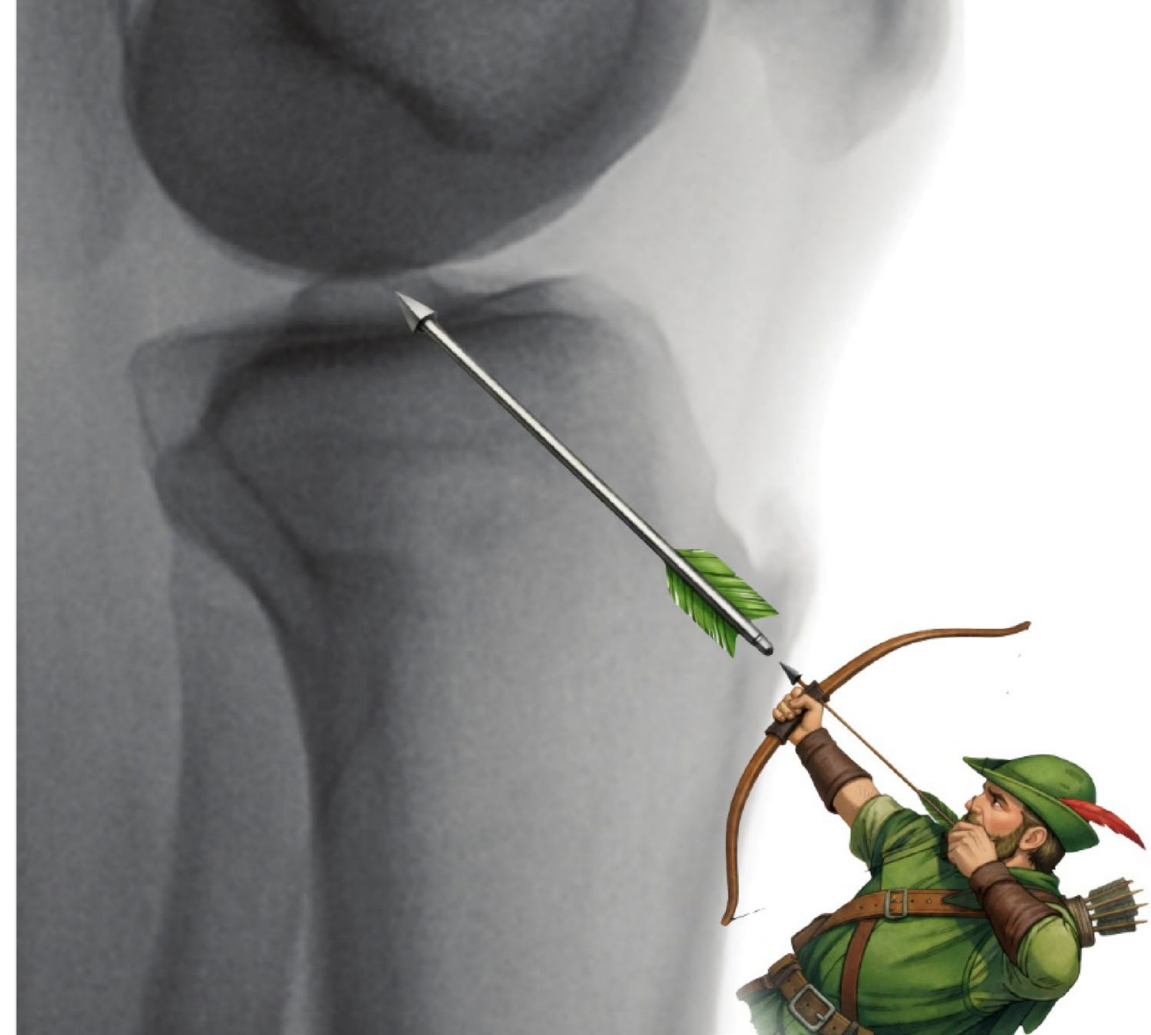
Schematic illustration of the Robin Hood technique. The entry point may be placed medial or lateral to the tibial tuberosity, with the trajectory directed toward the tibial spine, as indicated by the arrow. Illustration generated with the assistance of artificial intelligence (ChatGPT 5.2)

Correct screw positioning is then confirmed under fluoroscopy, and ACL stability testing is performed to assess knee joint stability (Fig. 1).

### Patients managed using the Robin Hood technique, as originally described by Frosch

#### Patient 1

**Fig. 2:**
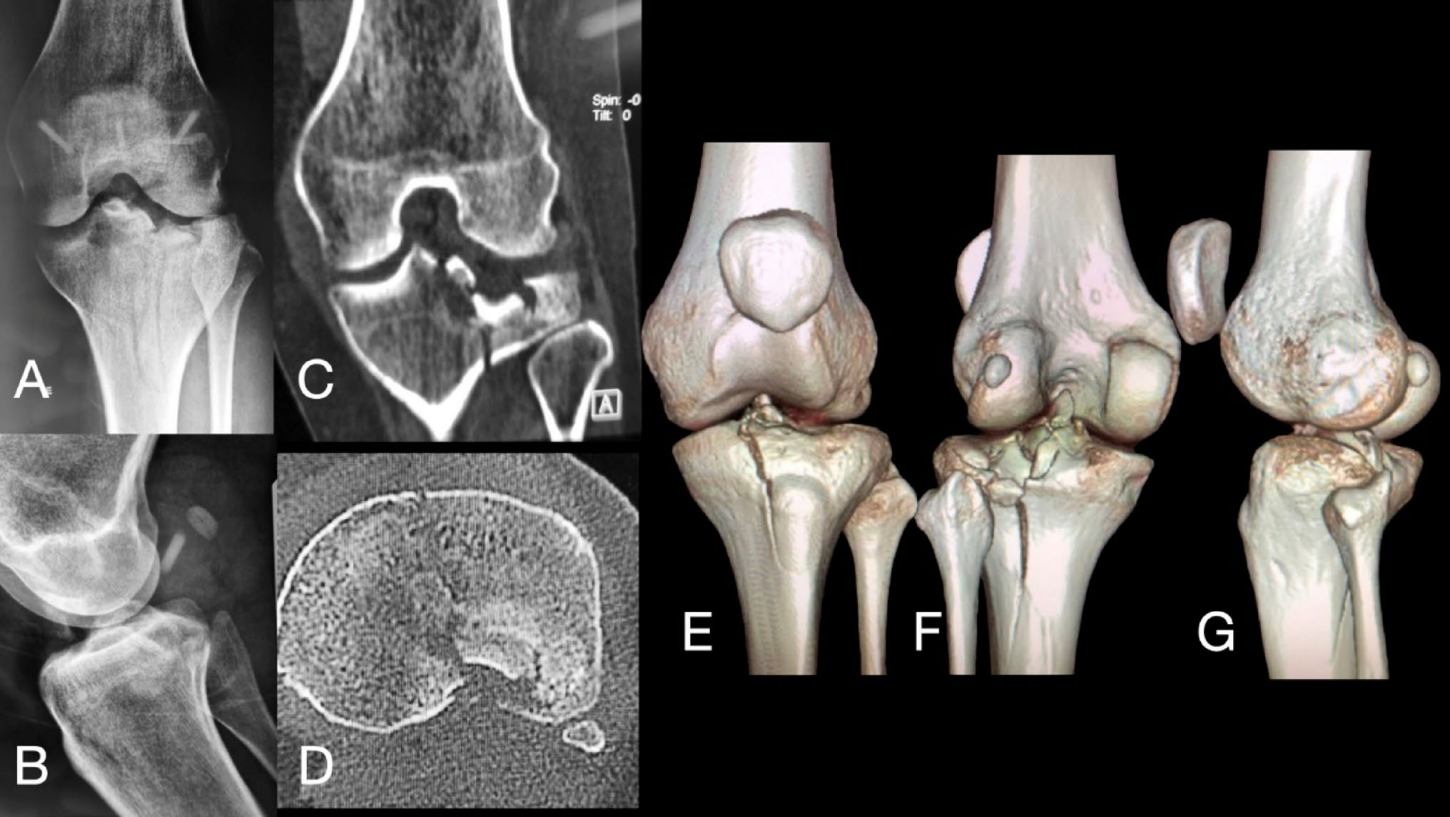
**A**, **B** Anteroposterior and lateral radiographs of the left knee demonstrating a tibial plateau fracture with posterolateral depression. **C**, **D** Coronal and axial computed tomography scans showing articular comminution and impaction of the posterolateral tibial plateau. **E–G** Three-dimensional CT reconstructions illustrating loss of the posterolateral rim of the tibial plateau, posterolateral articular depression, and an associated tibial spine avulsion fracture

A 31-year-old female patient sustained a motorcycle accident and presented with a closed fracture-dislocation of the left knee. Imaging evaluation demonstrated a tibial plateau fracture with an anteromedial coronal shear component and a posterolateral sagittal component, associated with posterolateral articular impaction and loss of the tibial plateau perimeter, in addition to an associated tibial spine fracture due to ACL avulsion (Fig. 2).

Transarticular spanning was not required, and the affected limb was placed in a plaster splint.

Four days after trauma, soft-tissue conditions were amenable for surgical management and the patient underwent open reduction and internal fixation of the tibial plateau and tibial spine fractures.

**Fig. 3:**
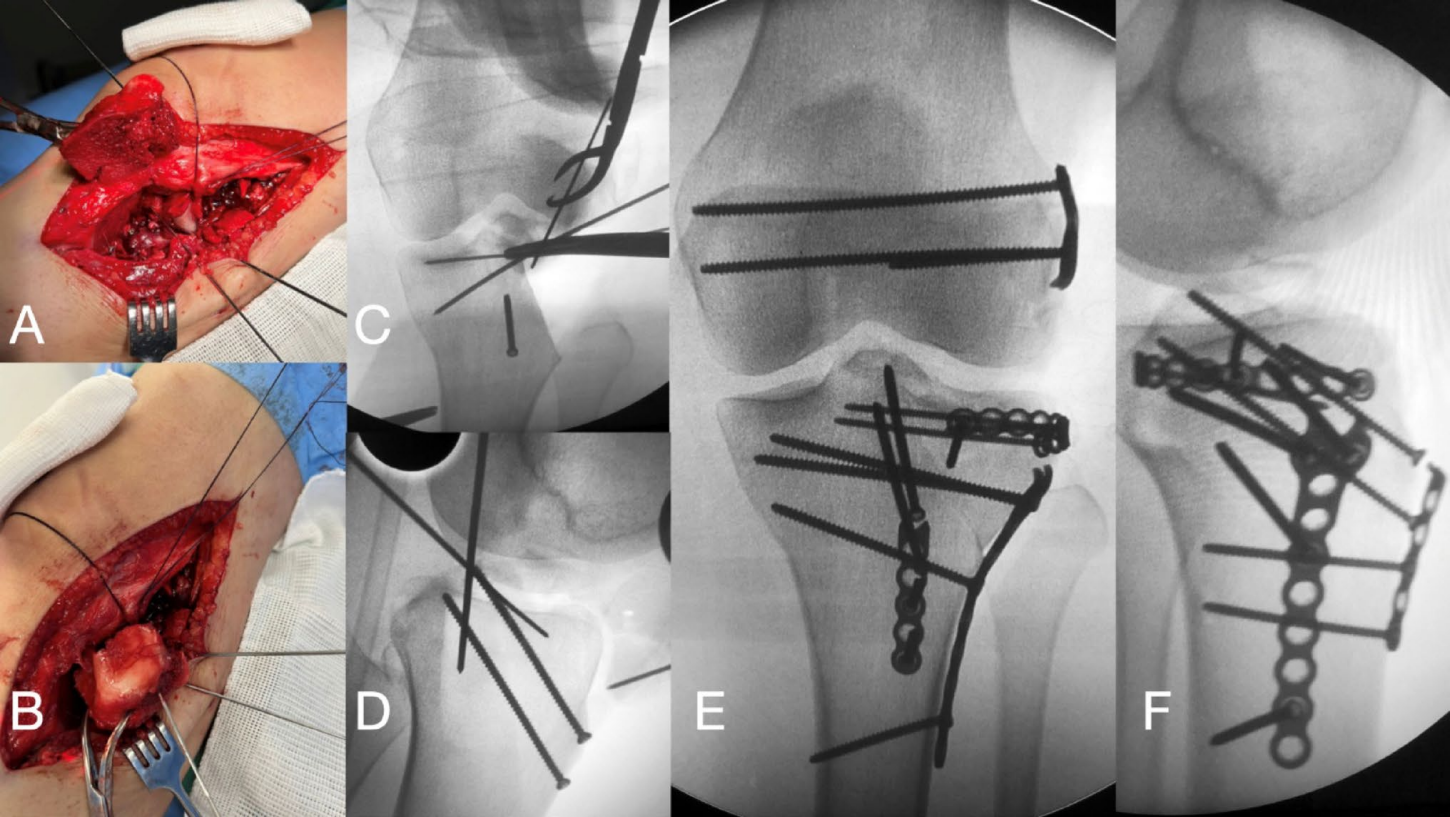
Intraoperative photographs of the anterolateral approach to the knee. **A** Osteotomy of the lateral femoral epicondyle and intra-articular osteotomy of the tibial plateau. **B** Reduction of the posterolateral articular depression with provisional fixation using Kirschner wires. **C** Intraoperative fluoroscopic image demonstrating reduction of the tibial spine fracture using a Kelly clamp. **D** Fixation of the tibial spine fracture with a 2.7-mm cortical screw (superior screw – Smith & Nephew^®^) and fixation of the tibial tuberosity with a parallel 2.7-mm cortical screw (inferior screw – Smith & Nephew^®^). **E**, **F** Postoperative fluoroscopic images of the left knee in anteroposterior **E** and lateral **F** views, respectively, demonstrating fixation of the intra-articular tibial plateau osteotomy with an anterolateral antiglide plate (2.7-mm hook plate, EVOS Mini – Smith & Nephew^®^) combined with a horizontal rim minifragment plate (2.4-mm EVOS Mini – Smith & Nephew^®^). Note the fixation of the lateral femoral epicondyle osteotomy with a minifragment plate (2.7-mm hook plate, EVOS Mini—Smith & Nephew^®^)

Due to the complexity of the plateau fracture, extended anterolateral approach with combined lateral femoral epicondyle and intra-articular tibial plateau osteotomies were required to reduce the posterolateral articular impaction (Fig. 3).

Postoperative rehabilitation included early mobilization for range-of-motion recovery and gradual weight-bearing progression, according to patient tolerance and radiographic signs of fracture healing.

**Fig. 4:**
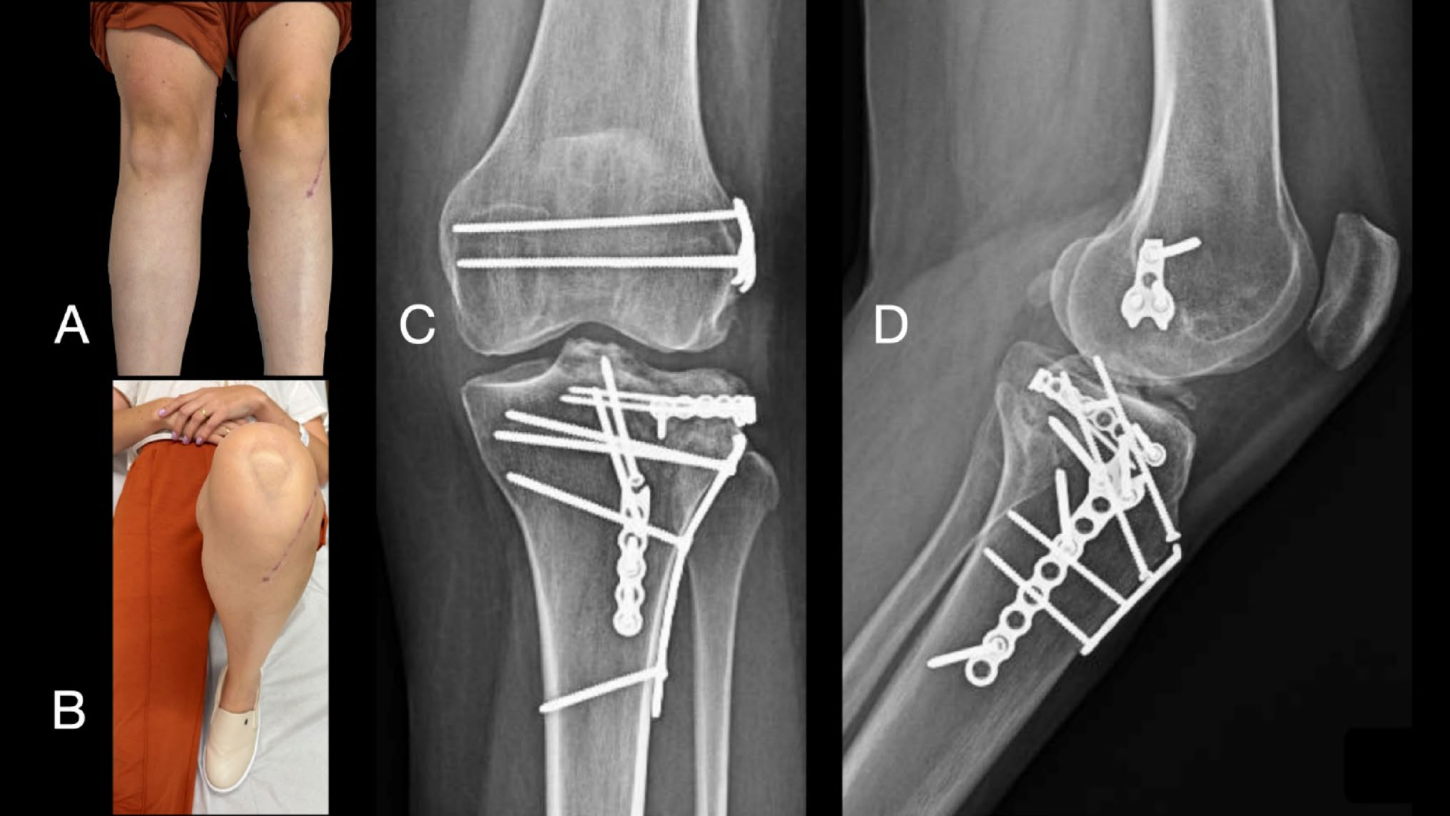
**A**, **B** Clinical photographs at 29 months postoperatively demonstrating full knee range of motion. **C**, **D** Anteroposterior and lateral radiographs of the left knee showing fracture healing in an appropriate alignment

At 29 months of follow-up, the patient demonstrated a knee range of motion symmetrical to the contralateral side, with a stable and painless joint, and had returned fully to pre-injury activities (Fig. 4). The patient achieved a KOOS (Knee Injury and Osteoarthritis Outcome Score) of 92% [11]. 

#### Patient 2

A 35-year-old female patient sustained a motorcycle accident resulting in a closed fracture of the diaphyseal and proximal metaphyseal regions of the tibia with articular extension. There were no signs of compartment syndrome or neurovascular injury.

The patient underwent imaging evaluation and immediate transarticular external fixation.

**Fig. 5:**
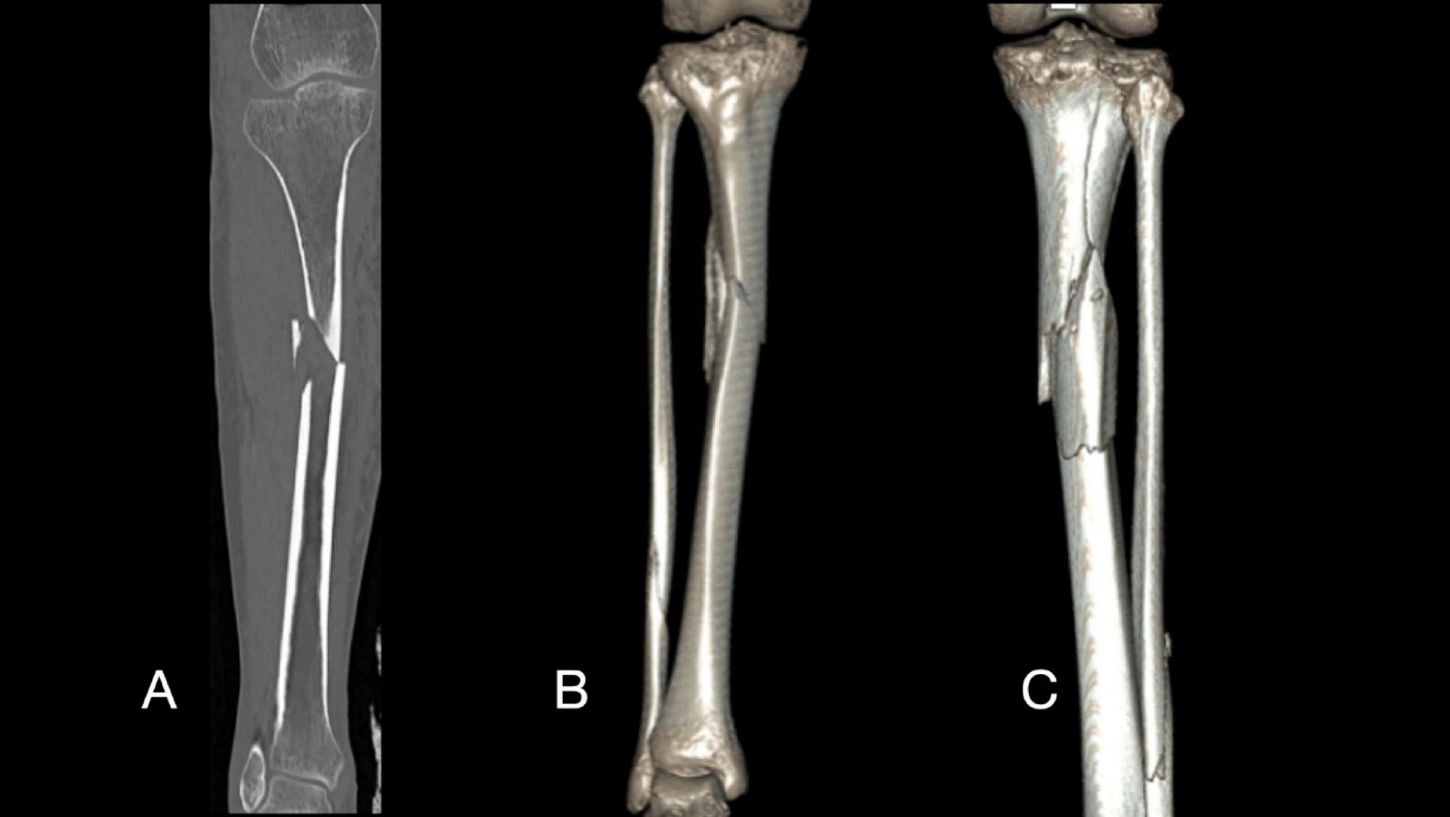
Coronal computed tomography (CT) scan (**A**) and three-dimensional reconstructions (**B**: anterior view; **C**: posterior view) of the leg. Note the intra-articular extension and the presence of a Meyers and McKeever type I tibial spine fracture, as modified by Zaricznyj [1]

Computed tomography revealed a tibial diaphyseal fracture with articular extension to the tibial plateau, in addition to a tibial spine avulsion fracture (Fig. 5).

**Fig. 6:**
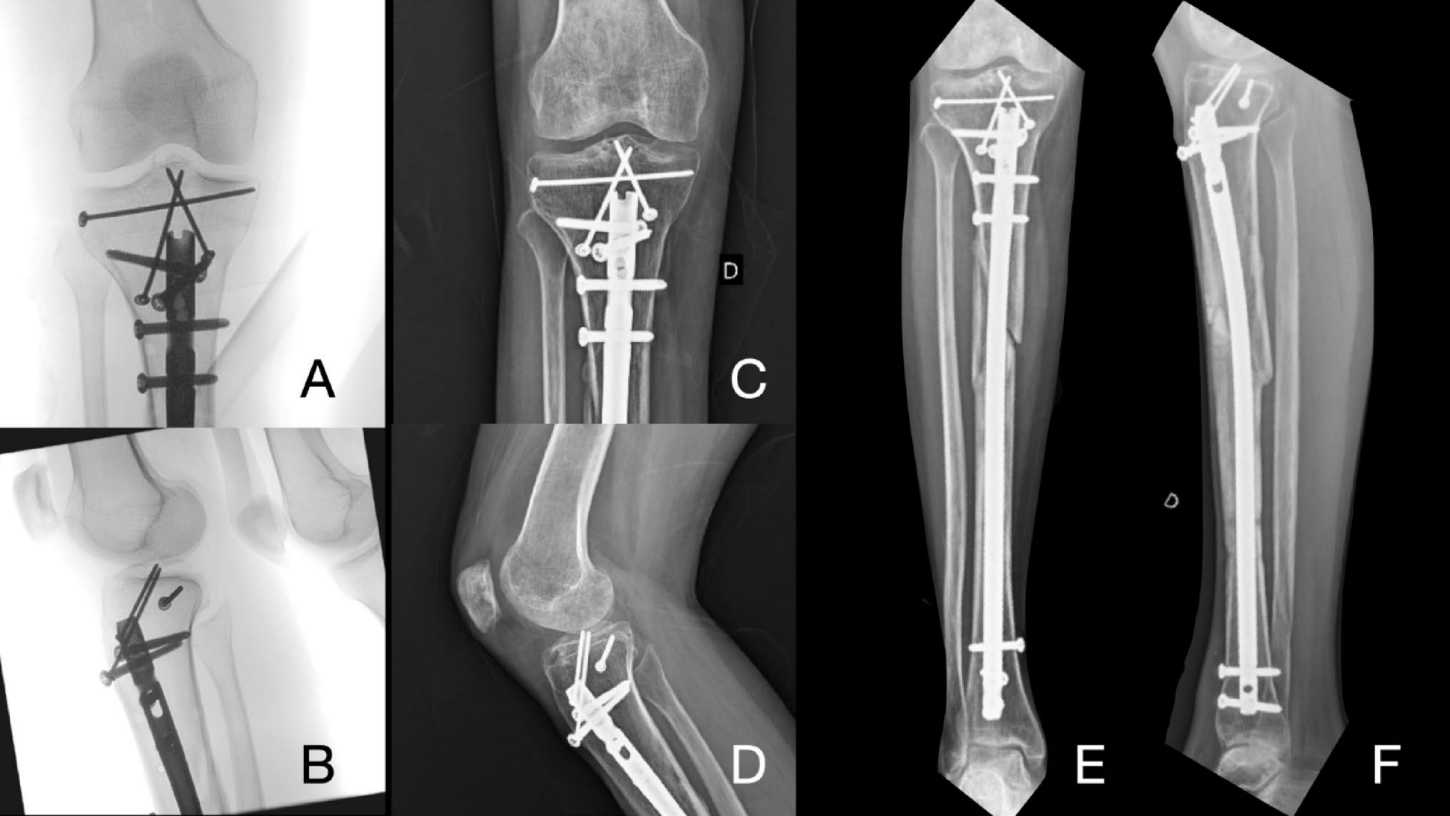
**A**, **B** Intraoperative fluoroscopic anteroposterior and lateral views of the knee demonstrating fixation of the tibial spine fracture with convergent retrograde screws, fixation of the sagittal tibial plateau component with a lag screw, and fixation of the diaphyseal component with a locked intramedullary nail. **C**, **D** Anteroposterior and lateral radiographs of the right knee obtained five months postoperatively, demonstrating union of the tibial spine fracture. **E**, **F** Anteroposterior and lateral radiographs of the right leg at five months showing bridging callus along the lateral cortex (AP view) and along the anterior and posterior cortices (lateral view), consistent with near-complete fracture healing

Eight days after external fixation, soft-tissue conditions were amenable for definitive internal fixation. The patient underwent fixation of the diaphyseal component with a suprapatellar intramedullary nail (ETN—DePuy Synthes^®^), fixation of the sagittal tibial plateau component with a 2.7-mm lag screw and washer (Smith & Nephew^®^), and fixation of the tibial spine avulsion fracture with two 2.7-mm cortical convergent screws (Smith & Nephew^®^) (Fig. 6).

The rehabilitation protocol included immediate initiation of knee range of motion and progressive weight bearing according to pain tolerance and radiographic progression of fracture healing. The patient developed fracture related infection at the incision used for reduction of the diaphyseal component, presenting with seropurulent discharge and local inflammatory signs 19 days after definitive fixation. She subsequently underwent removal of the minifragment plate used for provisional reduction, along with irrigation and debridement, microbiological sampling for culture and sensitivity testing, and a 30-day course of targeted antibiotic therapy. A debridement, antibiotics, and implant retention (DAIR) strategy was adopted, maintaining the intramedullary nail in situ. Enterobacter cloacae sensitive to ciprofloxacin was identified. The patient progressed with infection control and appropriate soft tissue healing, achieving a KOOS [10] score of 75% at six months of postoperative follow-up.

#### Patient 3

**Fig. 7:**
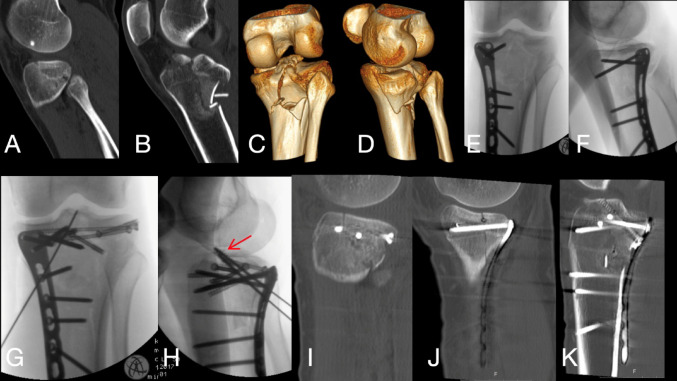
Computed tomography images in sagittal views **A**, **B** and three-dimensional reconstructions **C**, **D** demonstrate a tibial plateau fracture consistent with a flexion-type injury mechanism. The surgical procedure was completely perfomed in prone position. The operation was started by a posteromedial approach with a reduction and fixation of the main posteromedial fragment by buttress plate osteosynthesis (**E**, **F**). The bony avulsion of the PCL was reduced and fixated by two screws (**G**). The bony ACL avulsion was reduced by an ACL drill guide, which was introduced from posterior between ACL and PCL. The fragment was reduced with the drill guide, which had a sharp tip to reduce the fragment. Two guide wires from posterior were then drilled retrograde in the fragment (**H**). Two cannulated screws were inserted retrograde to fix the fragment (**H**, red arrow). The posterolateral fragment was reduced by using the posteromedial approach. After elevation of the posterolateral fragment with a chisel, the articular surface was supported by an allograft bone block (**I**). Fixation was performed with 3 cannulated, percutaneous screws in #-technique. Postop CT scan reveals an anatomical reduction of the articular surface with a proper fixation of the fracture (**I**–**K**)

A 28-year-old female patient suffered a flexion type tibial plateau fracture during skiing with a posteromedial coronal split fracture, an impression of the posterolateral tibial plateau and a bony tibial ACL and PCL (posterior cruciate ligament) avulsion (Fig. 7).

Surgical treatment consisted of open reduction and internal fixation of the posteromedial coronal fragment using a posterior buttress plate. After restoration of the proximal tibial alignment, the ACL tibial spine avulsion was anatomically reduced and fixed with screws inserted from posterior to anterior/distal to proximal direction. The associated PCL avulsion was addressed with independent screw fixation. Postoperative CT confirmed satisfactory reduction of both the articular surface and the tibial spine fragment (Fig. 7).

Postop rehab protocol included partial weight bearing for 6 weeks, continuous passive motion, and limited range of motion up to 90° for the same period. After 9 months the patient was completely free of symptoms with no limitations (Lysholm score was 100 points). Implant removal was not necessary [12]. 

#### Patient 4

A 40-year-old male patient sustained a fall from a scooter at a skatepark, resulting in a flexion-varus type tibial plateau fracture extending lateral to the intercondylar eminence (Wahlquist type C) with substantial joint depression [13]. 

**Fig. 8:**
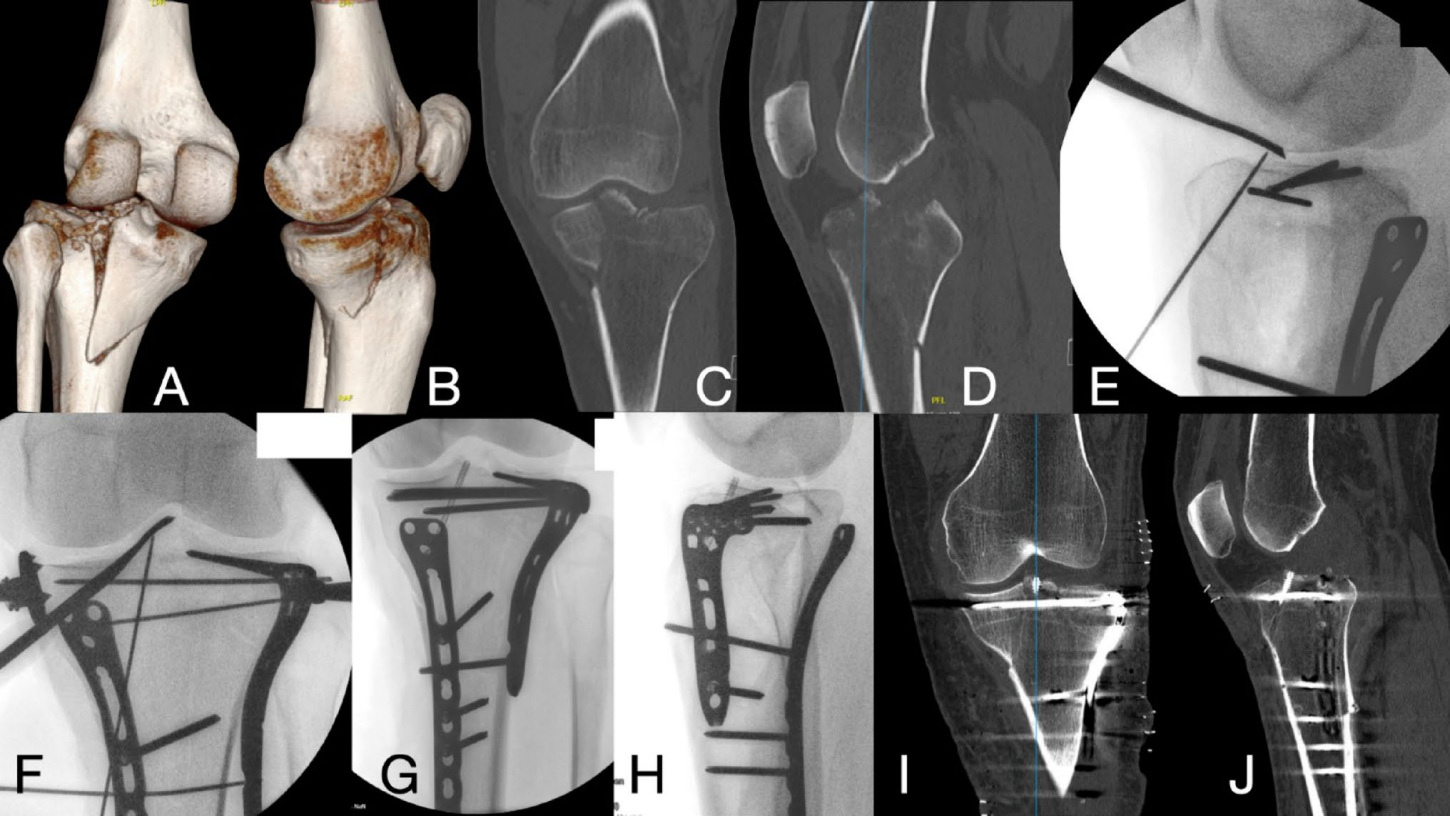
Computed tomography images, including three-dimensional reconstructions (**A**, **B**), coronal (**C**) and sagittal (**D**) views, revealed a flexion-varus type tibial plateau fracture with a bony avulsion fracture of the ACL. The patient was positioned supine, and surgery began with an anterolateral approach. A lateral tibial condyle osteotomy was performed to expose the depressed joint surface and lateral meniscus. There was no meniscal entrapment or tear, and the posterior root was intact. The lateral joint surface was reconstructed using two 2.7-mm subchondral screws and the osteotomized condyle was reduced back into position. Next, a posteromedial approach was performed, followed by buttress plating to ensure proper alignment. An anteromedial infrapatellar stab incision allowed the introduction of a small raspatory to reposition the ACL avulsion fragment, which was temporarily fixed with a K-wire (**E**). Interfragmentary compression was achieved after application of a lateral VA-LCP, restoring the condylar width (**F**). A second K-wire was used to guide placement of a 4 × 48 mm cannulated screw for definitive fixation of the ACL avulsion fracture (**G**, **H**). The knee was found to be stable both osseous and ligamentous on intraoperative assessment. Postoperative CT imaging demonstrated adequate fracture reduction and secure fixation of the ACL avulsion fragment (**I**, **J**)

The patient was positioned supine, and surgical treatment was performed through combined posteromedial and anterolateral approaches. The posteromedial and anterolateral fracture components were fixed with buttressing plates. Following restoration of the articular surface, the tibial spine fragment was reduced under fluoroscopic guidance through a limited anteromedial arthrotomy using a small raspatory. Definitive fixation was achieved with a 3.5-mm cannulated screw inserted using the Robin Hood technique. Postoperative imaging confirmed anatomical reduction of the intercondylar eminence and satisfactory restoration of the tibial plateau morphology (Fig. 8).

A postoperative hinged brace was applied with temporary flexion restriction to off-load stress on the ACL.

#### Patient 5

**Fig. 9:**
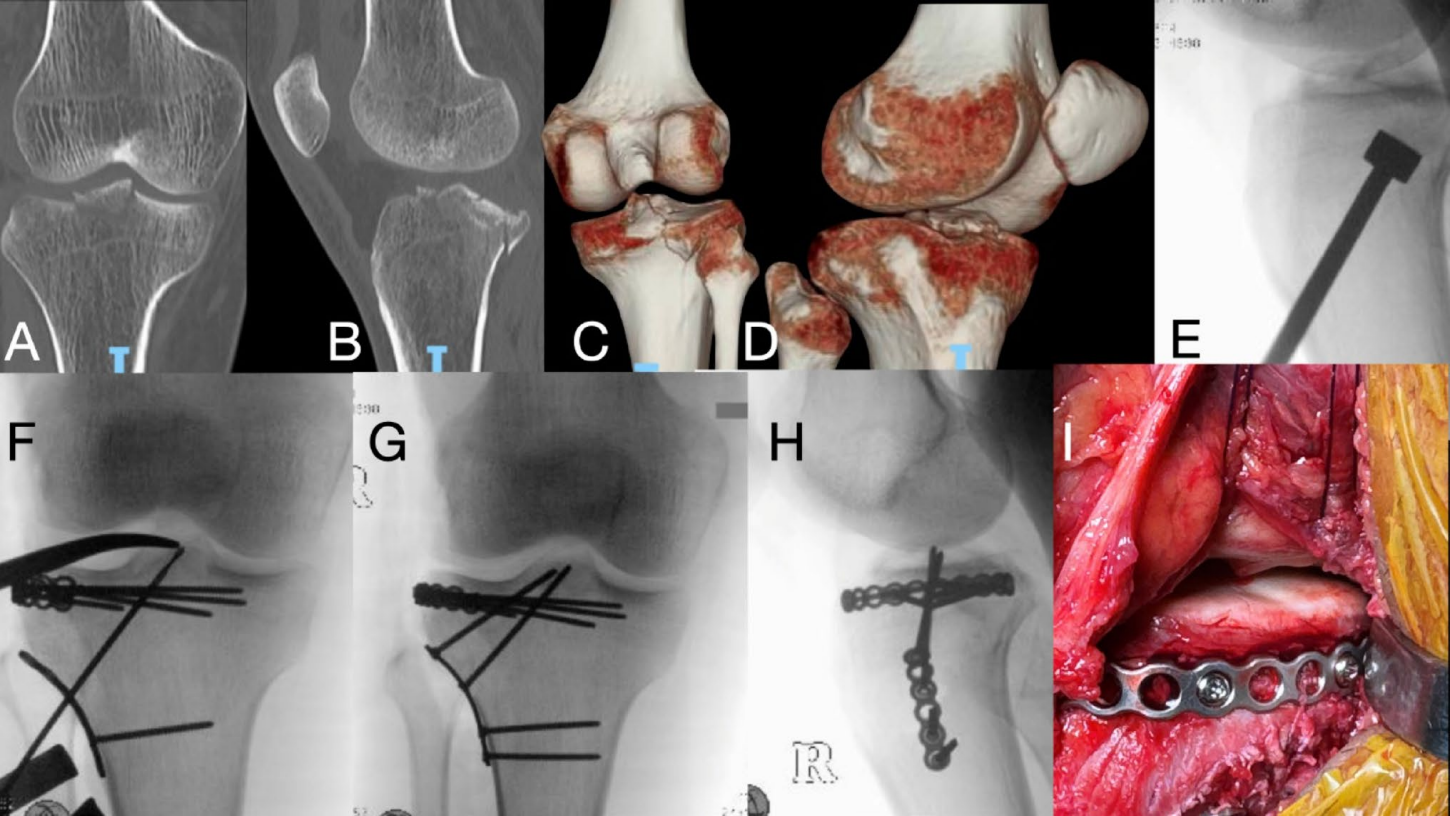
**A**, **B** Coronal and sagittal CT scans showing the tibial spine avulsion fracture associated with a posterolateral tibial plateau depression fracture. **C**, **D** Three-dimensional CT reconstructions demonstrating loss of the posterolateral tibial plateau rim. **E** Through an extended anterolateral approach, a cortical window was created in the lateral tibial plateau to allow insertion of a bone impactor, which was used to elevate and reduce the posterolateral depression. A 2.7-mm rim plate was applied through the anterolateral para-collateral fibular interval to contain the posterolateral rim, while rafting screws were used to support the reduced articular surface. A second 2.7-mm plate was placed to fix the cortical window and reduce the risk of a lateral tibial plateau cortical fracture. **F** Intraoperative fluoroscopic image demonstrating reduction of the tibial spine fragment using a Kelly clamp and the trajectory of the 2.0-mm drill directed toward the tibial spine for subsequent screw fixation. **G**, **H** Fluoroscopic images demonstrating anatomic reduction and final fixation. **I** Intraoperative photograph of the anterolateral approach demonstrating anatomic reduction of the articular surface

A 28-year-old previously healthy male sustained a tibial plateau fracture following a motorcycle accident. The injury consisted of a tibial spine avulsion associated with a posterolateral articular depression and loss of the articular rim. After soft-tissue conditions had adequately improved, the patient underwent open reduction and internal fixation 8 days after the injury. Note the reduction maneuver and fixation of the tibial spine fracture using two screws (Fig. 9).

The postoperative course was uneventful, with complete restoration of knee range of motion achieved early in the postoperative period.

## Discussion

The name of the technique described by Karl-Heinz Frosch is inspired by the character Robin Hood, popularized by the American writer and illustrator Howard Pyle in The Adventures of Robin Hood. This character is traditionally associated with exceptional accuracy in striking a precise target, a concept that parallels the technical objective of achieving accurate, targeted fixation.

Analogously, the method is based on introducing one or more screws in a retrograde direction, from distal to proximal and from anterior to posterior or posterior to anterior, with a carefully planned trajectory directed toward the tibial spine.

Based on the available literature indexed in major databases (PubMed, EMBASE, Web of Science), we did not identify a similar published technical description of this specific configuration for tibial spine fixation.

In tibial plateau fractures, central comminution may occur in up to approximately 30% of cases, involving tibial spine region [14]. In a retrospective cohort of 122 adult patients treated with internal fixation, predominantly bicondylar patterns (67%) and high-energy mechanisms (84%), the mean displacement of the tibial spine fragment decreased from approximately 4 mm preoperatively to about 1 mm following plateau fixation. Early conversion to arthroplasty occurred in only 4% of cases. Specific fixation of the tibial spine was performed in a small proportion of patients (4%) and was not associated with measurable improvement in the evaluated outcomes.

Although earlier literature suggested uncertainty regarding the indications for fixation of tibial spine or ligamentous avulsion fragments in tibial plateau fractures, more recent biomechanical and clinical evidence supports fixation in specific injury patterns [15, 16]. In posterolateral tibial plateau fractures associated with ACL injury, increased tibial plateau displacement and ligament strain have been demonstrated, supporting reduction and fixation to restore knee stability [15, 16]. Similarly, flexion-varus tibial plateau fractures show a strong association with ACL avulsion injuries, highlighting the importance of addressing bony avulsion fragments to prevent persistent instability and meniscal pathology [6]. Collectively, these findings indicate that fixation of associated bony avulsion fragments should not be regarded as optional in selected tibial plateau fracture patterns, but rather as an essential component of restoring knee stability and preventing secondary instability or poor functional outcomes.

This technical note has inherent limitations related to its descriptive design, including a small sample size and the absence of a control group, which precludes any inference regarding superiority, equivalence, or clinical impact when compared with alternative fixation strategies or with non-fixation in cases of non-displaced or displaced tibial spine fractures. It is not intended as an efficacy study, but rather as a description of a surgical technique and its underlying rationale applied to a specific injury pattern. Furthermore, the reproducibility and generalizability of these findings require validation in larger clinical series and comparative studies. An additional limitation of this study is the use of different instruments for functional outcome assessment (i.e. KOOS and Lysholm score) [11, 12]. This variability arises from the inclusion of patients treated across different centers, each adhering to its own standardized postoperative evaluation protocol, and may limit the comparability of functional outcomes.

Nevertheless, the relevance of this technical note lies in its detailed description of a retrograde fixation configuration for the management of combined tibial spine and proximal tibial metaphyseal fractures. By describing the surgical technique and its application, this report expands the available surgical armamentarium and provides an additional option to support intraoperative decision-making.

## Conclusion

The “Robin Hood” technique represents an alternative retrograde fixation for tibial spine fractures associated with proximal tibial metaphyseal fractures. As a technical note based on the description of five cases, its findings do not allow for conclusions regarding comparative efficacy or for recommendations of routine use. However, the technique demonstrates technical feasibility and practical applicability within the described clinical context and may be considered an additional option in surgical planning.

## Data Availability

No datasets were generated or analysed during the current study.
